# Non-linear rheology reveals the importance of elasticity in meat and meat analogues

**DOI:** 10.1038/s41598-021-04478-z

**Published:** 2022-01-25

**Authors:** Floor K. G. Schreuders, Leonard M. C. Sagis, Igor Bodnár, Remko M. Boom, Atze Jan van der Goot

**Affiliations:** 1 Laboratory of Food Process Engineering, Wageningen University, Bornse Weilanden 9, 6708 WG Wageningen, The Netherlands; 2 Laboratory of Physics and Physical Chemistry of Foods, Bornse Weilanden 9, 6708 WG Wageningen, The Netherlands; 3 Firmenich S.A, Rue de la Bergére 7, 1242 Geneva, Satigny Switzerland

**Keywords:** Rheology, Polymers

## Abstract

The interest in plant-based meat analogues as an alternative to meat is currently growing. Rheological benchmarking is used to reveal how closely meat analogues resemble the original meat products. Texture maps and dissipation colour schemes were used to reveal similarities in and differences between rheological responses of meat and meat analogues (especially chicken analogues). Under heating, meat analogues differ in terms of their lower elasticity compared with heated meat. The changes caused by heating meat and meat analogues were different as well. Heating of meat resulted in a tougher and more elastic material, while heating has a minor effect on meat analogues. Future developments should therefore focus on routes to create more elasticity and possibly allow heating effects on texture to mimic meat characteristics even better.

## Introduction

Meat analogues are designed to have sensory properties similar to meat but are made from plant proteins^1^. The unique textural properties of meat are a result of the muscle tissue structure consisting of repeating sarcomere structures that are grouped into muscle fibres and surrounded by connective tissue, the cell membrane, and, depending on the meat type, stored intramuscular fat tissue^2,3^. Fish muscle is mainly composed of myotomes, which are muscular sheets connected to one another by connective tissues, called myocomnata. Myotomes are mainly composed of myosin and actin mainly that form large numbers of muscle fibers^4^.

Food scientists have mimicked fibrous meat and fish-like structures with plant-based proteins through high moisture extrusion cooking and shear cell technology^5,6^. Meat analogues often contain blends of textured and non-textured proteins from soy, wheat or pea, which after processing can be used to create a range of morphologies^7,8^. Some meat analogue products aim at mimicking whole-cut meats, like chicken meat, pork and beef steak, which are characterized by the presence of long fibres or layers. The structure characteristics of meat have been widely studied. However, methods that quantitatively compare meat and meat analogues are not widely available^9^. It was recently found that a closed-cavity rheometer developed for the rubber industry allows accurate determination of the rheological properties of concentrated plant-based protein materials^5,6,10,11^. In a recent study, single ingredient dispersions of soy, pea and gluten as well as pea-gluten and soy-gluten blends were studied with this device^12,13^. A combination of texture maps and dissipation colour schemes was used to map the properties of the ingredients and the blends. The dissipation ratio as a function of small and large strain amplitudes was useful for understanding the differences in elasticity. Heating of plant protein blends showed an increase in the elasticity. Here we extend rheological measurements to commercial meat and meat analogue products, and especially those that can be considered as whole-cut products.

The objective of the current study is therefore to quantify the similarities and differences between meat and meat analogues. The stresses at the ends of the LVE regime and the stress–strain crossover points are captured in texture maps. The energy dissipation ratio is represented by colour contours in strain-temperature diagrams. For the design of plant-based meat analogues, the maps and schemes may guide the design and selection of plant-based materials to be as similar as possible to meat.

## Materials and methods

### Materials

Meat and meat analogues were bought from a local supermarket (Albert Heijn) in Wageningen, The Netherlands, which were then stored at 4 °C before further analysis within two days. Meat and fish samples used include chicken (more specifically the breast of a chicken), beef (Semitendinosus muscle), pork (Longissimus muscle), codfish and salmon. The meat analogues used are “Kipstuckjes” (Vegetarian Butcher), “Chick free pea based” (Nutrali Foods), “Stukjes als van Kip” (AH), “Vegetarische basis wokstukjes (AH)” and “Vivera plant stukjes als kip” (Vivera). The manufacturers’ specifications of the composition of meat and meat analogues are presented in Tables 1 and 2. The specific signatures product of meat analogues and ingredients according to the packaging are presented in Table 3.

**Table 1:** Composition of meat and fish.

	Chicken	Beef	Pork	Codfish filet	Salmon
Water	74.7	73.2	72.9	83.1	63.9
Protein	23.6	21.3	23.0	16.0	20.0
Fiber					
Carbohydrate					
Fat	1.6	5.3	4.0	0.7	16.0
Salt	0.1	0.2	0.1	0.2	0.1

**Table 2:** Composition of meat analogues.

	Kipstuckjes	Chick free pea based	Stukjes als van Kip	Vegetarische basis wokstukjes	Vivera plant stukjes als kip
Water	61.1	67.6	61.97	63.6	66.5
Protein	20.0	21.0	23.0	21.0	19.0
Fiber	7.6	2.8	0.2	5.5	5.6
Carbohydrate	5.0	5.8	6.5	4.0	6.7
Fat	4.4	1.7	7.0	4.5	0.5
Salt	1.9	1.1	1.3	1.4	1.7

**Table 3:** Description of meat analogues and ingredients according to the packaging.

Signature product	Company	Ingredients according to packaging	Website
Kipstuckjes	The vegetarian Bucher	88% soy structure (soy protein, water), spice-extract, sunflower oil, natural flavoring	https://www.thevegetarianbutcher.co.uk/
Chick free pea based	Naturli’ Foods	Water, texturized pea protein concentrate (31%), vegetable bouillon (salt, dextrose, yeast extract, dried vegetables, natural flavouring), fermented dextrose	https://www.naturli-foods.com/
Stukjes als van Kip	Albert Heijn	Rehydrated vegetable protein (soy, wheat), sunflower oil, free-run egg protein, wheat starch, vinegar, natural flavors, aroma, water, sea salt, salt, maltodextrin, iron, vitamin B12	https://www.ah.nl/
Vegetarische basis wokstukjes	Albert Heijn	Rehydrated vegetable protein (40% soy, 40% wheat), sunflower oil, free-run egg protein powder, wheat fiber, flavorings, wheat starch, natural flavor, thickener (carrageenan [E407]), diet salt (potassium chloride), dextrose, spices, maltodextrin, yeast extract, onion, salt, iron, vitamin B12	https://www.ah.nl/
Vivera Plantaardige kipstuckjes	Vivera	93% rehydrated soy protein, onion extract, natural flavors (contains wheat), pea fiber, salt	https://vivera.com/

### Rheological properties

The rheological properties were measured with a closed cavity rheometer (CCR) (RPA elite, TA instruments, New Castle, Delaware, USA)^14^. Approximately 6 g of product was placed between two plastic films in the cavity, sealed with a closing pressure of 4.5 bar to prevent water evaporation during heating. The closed cavity rheometer has a radius of 22.5 mm and the maximum height of the inner cavity is 4 mm having a biconical opening with an angle of 3.35° for ensuring a homogeneous shear field. Grooves on the surface of the cones prevent slip. The lower cone oscillates in strain-controlled mode while the upper cone remains stationary. In a first test, the meat and meat analogues were heated to 30 °C without shear shearing for 2 min. Strain sweep (0.01–1000%, using a frequency of 1 Hz) experiments were performed at 30 °C. In a second test, the meat and meat analogues were heated to 65 °C without shear shearing for 2 min. Strain sweep (0.01–1000%, constant frequency at 1 Hz) experiments were performed at 65 °C. The latter temperature is often considered as the desired core temperature when preparing red meat. In a third test, the meat and meat analogues were heated at 65 °C for 2 min and cooled to 30 °C with a cooling rate of 5 °C/min without a shear treatment. Strain sweep (0.01–1000%, constant frequency at 1 Hz) experiments were performed when cooled down to 30 °C.

Strain sweep experiments were used to determine the yield stress and the flow stress of a material. The yield stress is defined here as the value of the shear stress at the end of the LVE regime. Here we define this stress as the point where ${G}^{{{\prime}}}$ differs more than 5% from its strain-independent value in the LVE regime^15,16^. The flow stress is defined here as the value of the shear stress at the crossover point, where the storage modulus is equal to the loss modulus (${G}^{{{\prime}}}={G}^{"})$.

### Large amplitude oscillatory shear (LAOS)

The stress and strain data obtained from the LAOS measurements were analysed using the MITlaos software (Version 2.1 beta, freeware distributed from MITlaos@mit.edu), using a similar protocol as reported by Schreuders et al.^12^. The strain amplitude was varied in the range of 0.01–1000% at a constant frequency of 1 Hz at 30 °C. Lissajous curves were used to relate the response of the protein materials to the imposed oscillatory strain.

The area enclosed by a Lissajous curve can be interpreted as the energy dissipated per unit volume during one complete cycle of the imposed oscillatory strain. The energy dissipated per unit volume in a single cycle (${E}_{d}$) is a function of the first-order viscous Fourier coefficient (${G}_{1}^{"}$; calculated from the intensity and phase of the first-harmonic) only (Eq. (1)):

1 $${E}_{d}=\oint \sigma d\gamma = \pi {{G}_{1}^{"}\upgamma }_{0}^{2}$$

The energy dissipated by a perfectly plastic material in a single cycle (${({E}_{d})}_{pp}$) is equal to Eq. (2)

2 $${({E}_{d})}_{pp} =4 {\upgamma }_{0}{\sigma }_{max}$$

for a given strain amplitude (${\upgamma }_{0}$) and maximum stress (${\sigma }_{max}$). The ratio of the actual dissipated energy and the perfectly plastic dissipation gives the energy dissipation ratio *(*$\varphi$) as proposed by Ewoldt et al.^17^ (Eq. (3)).

3 $${\upvarphi }=\frac{{E}_{d}}{{{(E}_{d})}_{pp}}=\frac{\pi {G}_{1}^{"}{\upgamma }_{0}}{4{\sigma }_{max}}$$

### Texture maps and dissipation colour schemes

**Figure 1:**
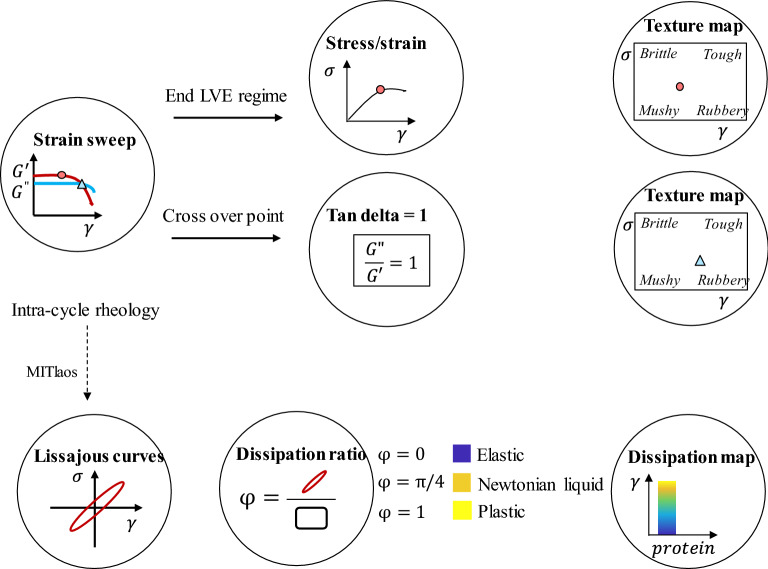
The storage modulus (${G}^{{{\prime}}}$) and loss modulus (${G}^{"}$) versus strain to define the crossover point (${G}^{{{\prime}}}={G}^{"})$ and the end of the linear viscoelastic (LVE) regime^16^.

Texture maps summarize rheological information into two-dimensional plots. Here the texture maps are based on the stress and strain at the end of the linear viscoelastic (LVE) regime and the stress and strain at the crossover point, to classify products into four quadrants (Fig. 1)^18^. Materials in quadrant 1 (Fig. 1 lower left), with low shear stress and low shear strain, were classified as a soft, non-shaped texture commonly referred to as “mushy” (such as for grits and similar food materials). Materials in quadrant 2 (Fig. 1 lower right), with low shear stress and high shear strain, are often described as “rubbery” (such as gelatin). Quadrant 3 (Fig. 1 top right), indicating strong materials with high shear stress and shear strain, indicates a “tough” texture (for example, fruit leather and dried fruits). Quadrant 4 (Fig. 1 top left), with high shear stress and low shear strain, characterises products with a “brittle” texture (such as many baked or confectionery food products^19^.

The energy dissipation ratio is depicted by colour contours in strain–temperature diagrams. When $\varphi =0$ (blue), the rheological response is purely elastic, while $\varphi =1$ (yellow) implies that the material is a perfect plastic^20^. A $\varphi$ of π/4 corresponds to a material that behaves as a Newtonian liquid.

### Statistics

The strain sweep experiments were performed in duplicate. Means and standard deviations were calculated using SPSS (Version 22.0, IBM, USA) statistical software. One-way ANOVA (Duncan's test at a 95% confidence level) was performed to evaluate the statistical significance between samples. LAOS tests were performed only once per sample, but as a results, no additional statistical analysis can be performed on LAOS samples.

## Results and discussion

### Texture maps of meat and meat analogues

**Figure 2:**
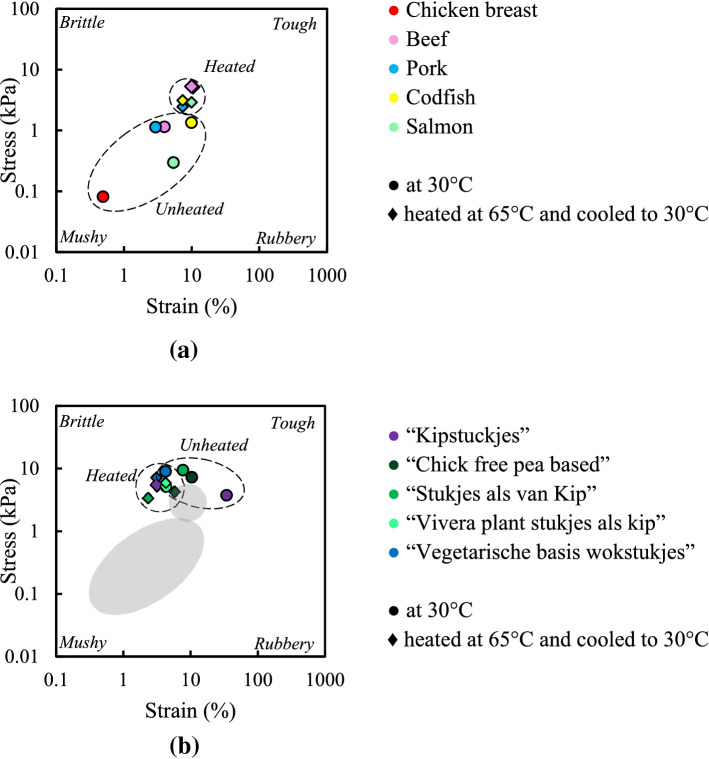
Texture map at the end of the LVE regime for meat (**a**) and meat analogues (**b**) heated at 30 °C and heating to 65 °C, and cooling to 30 °C. Lines are drawn to guide the eye and shows the outline. The grey area in the texture map of meat analogues shows the outline of meat products, shown also in the (**a**).

Figure 2 presents a texture map using the stress and strain values at the end of the LVE regime of meat and meat analogues at 30 °C, and samples heated at 65 °C and subsequently cooled to 30 °C. At 30 °C, meat products behave “mushy” whereas meat analogues are “tough”. Heating and subsequent cooling (i.e. at 65 °C and cooled to 30 °C) of meat products shifted the texture from mushy to tough. This behaviour was most pronounced for chicken and salmon. Heat-denaturation of myofibrillar proteins in meat generally caused toughening^21^. After heating, the meat products (e.g. chicken and codfish) were found to be in the same area of the map as the meat analogues (e.g. “Kipstuckjes” and “Chick free pea based”) (Table S1). For meat analogues (e.g. “Vivera”) there was only a small shift due to a heat treatment observed in the texture maps, meaning that stress and strain values at the end of the LVE were hardly influenced by heating (Table S1). Some meat analogues slightly shift into the opposite direction (“tough” to “mushy”), as compared with meat. The limited effect of heating on meat analogues is not unexpected since the commercial meat analogues tested were most likely heated during processing and any denaturation or crosslinking of protein had been completed already as a result of that thermal treatment.

**Figure 3:**
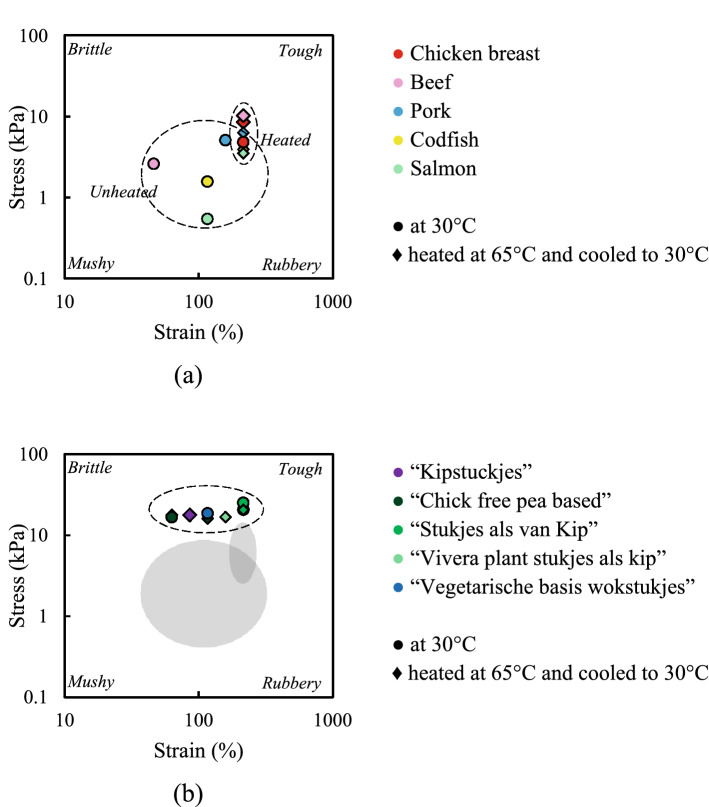
Texture map at the crossover point for meat (**a**) and meat analogues (**b**) heated at 30 °C and heating to 65 °C, and cooling to 30 °C. Note that the scales of the x and y axes are different from those in Fig. 2. Lines are drawn to guide the eye and shows the outline, the grey area in the texture map of meat analogues shows the outline of meat products shown in (**b**).

The end of the LVE is not very “sensitive” to structural differences, and therefore we proceed to evaluating the behaviour at the crossover point in Fig. 3. This point is generally considered as the strains where the storage modulus is equal to the loss modulus (${G}^{{^{\prime}}}={G}^{"})$. Heating and cooling (i.e. at 65 °C and cooled to 30 °C) meat products gave a shift in the texture map from mushy to tough. The crossover stresses of different meat analogues at 30 °C and pre-heated at 65 °C and cooled to 30 °C are comparable but the crossover strains are different for each meat analogue (Table S2).

### Dissipation colour scheme to describe the dissipation ratio

**Figure 4:**
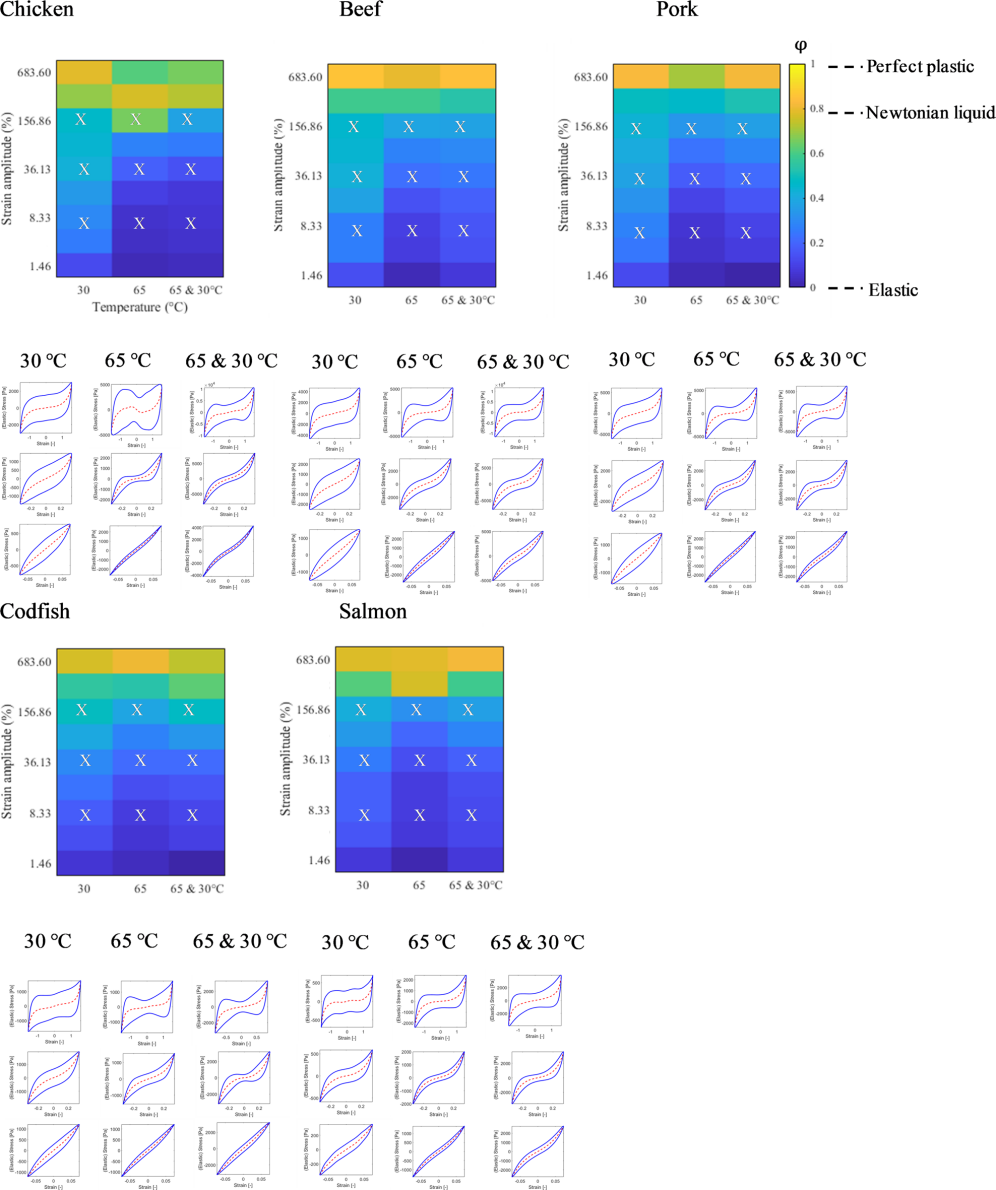
Colour scheme of the dissipation ratio (${\upvarphi }$) in a strain–temperature diagram for meat and fish at 30 °C, heated at 65 °C, and heated at 65 °C and cooled to 30 °C. The colour corresponds to the value of the dissipation ratio (${\upvarphi }$) in the colour bar, ${\upvarphi }$= 0, elastic; ${\upvarphi }\hspace{0.17em}$= π/4, Newtonian liquid; ${\upvarphi }$= 1, perfect plastic. Lower part: Lissajous curve of stress versus strain amplitude at three different strain amplitudes corresponding to the X symbol in the upper panel (individual plots of normalized stress [solid lines] and elastic stress [dashed lines] vs. strain).

**Figure 5:**
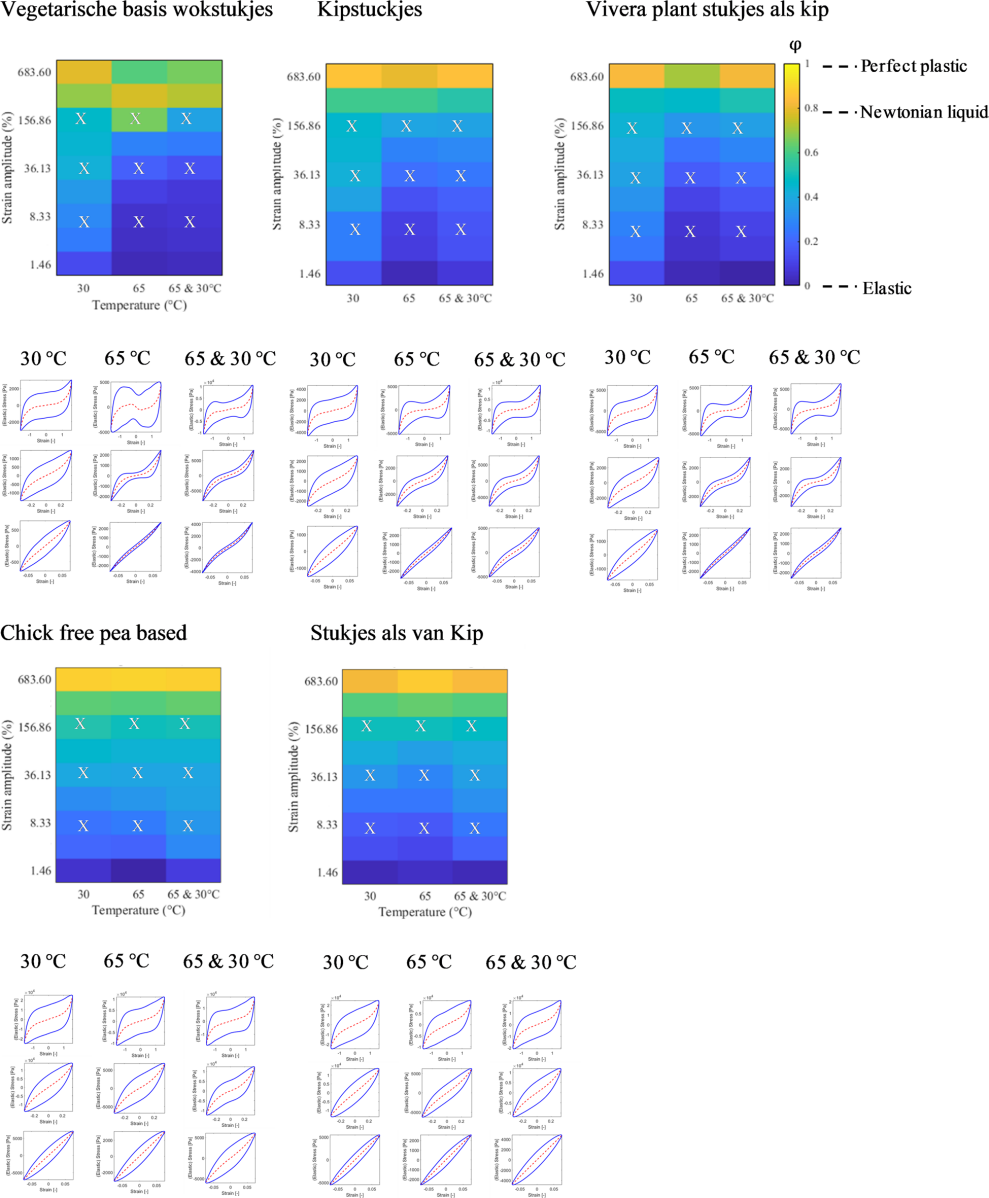
Colour scheme of the dissipation ratio (${\upvarphi }$) in a strain–temperature diagram for meat analogues at 30 °C, heated at 65 °C, and heated at 65 °C and cooled to 30 °C. The colour corresponds to the value of the dissipation ratio (${\upvarphi }$) in the colour bar, ${\upvarphi }\hspace{0.17em}$= 0, elastic; ${\upvarphi }$= π/4, Newtonian liquid; ${\upvarphi }\hspace{0.17em}$= 1, perfect plastic. Lower part: Lissajous curve of stress versus strain amplitude at three different strain amplitudes corresponding to the X symbol in the upper panel (individual plots of normalized stress [solid lines] and elastic stress [dashed lines] vs. strain).

A more detailed assessment of the non-linear viscoelastic behaviour of meat and meat analogues was obtained using Lissajous curves. The dissipation ratio was calculated to extract the essence of the non-linear behaviour^17,22–24^. The dissipation values are presented in a dissipation colour scheme to facilitate comparison. The upper panel of Figs. 4 and 5 show dissipation colour schemes for meat and meat analogues. The lower panel of Figs. 4 and 5 present the corresponding elastic Lissajous curves for the grid values of imposed strain amplitude and temperature marked by the crosses in the left-hand panel (dissipation colour scheme of the dissipation ratio). As expected, both meat and meat analogues show an increase in the dissipation ratio for increasing strain amplitudes, shown by an enlargement of the area encompassed by the Lissajous curves. Heating made the meat more elastic for all strain amplitudes, but a more sudden transition to viscous/plastic behaviour was observed as well.

For meat, at 30 °C, narrow ellipses were observed at low strain amplitude, indicating an increase in sample’s elasticity. At higher strain amplitudes the Lissajous curves showed a gradual transition to a more rhomboidal shape, indicating a transition to plastic behaviour. After heating to 65 °C, and cooling, the plots become more narrow than the plots of the unheated meat samples, indicating an increase of the elasticity in the sample. Compared with the unheated samples we now see a much stronger strain stiffening effect in the intermediate strain range, shown by an inverted sigmoidal shape of the plots. At the highest strains the sigmoidal shape widens, indicating a gradual transition to plastic behaviour. For codfish and salmon similar dissipation colour maps and Lissajous curves were found as for meat, including the observation that after heating, the curves attain an inverted sigmoidal shape.

Depending on the strain amplitude and products, darkening of the blue colour in maps indicates that the products gained elasticity. The positive effect of heating on elasticity was shown for two products (Vegetarische basis wokstukjes & Kipstuckjes) treated under low and mid-level shearing. Heating did not change ‘the shape’ of Lissajous curves for the case of two meat analogues products (Chick free pea-based & Stukjes als van Kip). Instead, the dissipation ratio for meat analogues transitioned from elastic to plastic gradually as a function of increasing strain amplitudes in a similar manner as unheated meat analogues.

**Figure 6:**
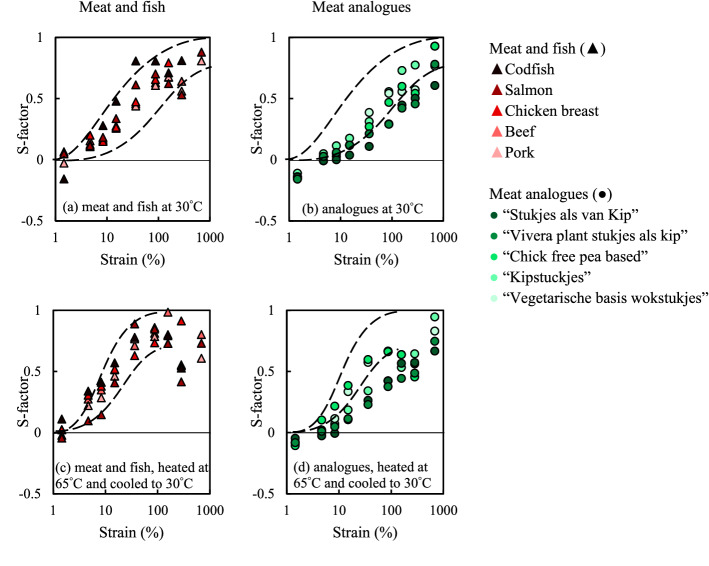
Stiffening ratio (S-factor) as a function of strain amplitude for meat and meat analogues. Dashed lines are drawn to guide the eye and show the outlines for the highest and lowest values of meat and fish from the meat analogues graphs.

To describe the local changes of the material inside one strain cycle, the stiffening ratio or S-factor, is introduced in Eq. (4):

4 $${\rm S}=\frac{{G}_{L}^{{\prime}}-{G}_{M}^{{\prime}}}{{G}_{L}^{{\prime}}}$$

where ${G}_{L}^{{\prime}}$ is the large strain (or secant) modulus, which describes the slope of a line between the origin and the stress at maximum strain in the Lissajous curve. The modulus ${G}_{M}^{{\prime}}$ is the slope of the Lissajous curve at zero strain. In the linear regime, ${G}_{L}^{{\prime}}$=${G}_{M}^{{\prime}}$, and therefore ${\rm S}$=0 by definition. In Fig. 6, the S-factor is plotted as a function of the strain amplitude. For small strain amplitudes, the S-factor is indeed close to zero. With increasing strain amplitude, the S-factor increases indicating intra-cycle strain stiffening. After heating to 65 °C and cooling, the meat products (i.e. chicken, pork, beef, salmon and codfish) exhibit a larger S-factor and thus a stronger strain stiffening effect in the intermediate strain range compared to the unheated samples. The meat analogues products clearly showed less strain stiffening upon deformation.

All materials show apparent stiffening; showing decreasing curves for the ${G}_{L}^{{\prime}}$, and ${G}_{M}^{{\prime}}$. For meat and fish, the ${G}_{M}^{{\prime}}$ decreases faster than the ${G}_{L}^{{\prime}}$ indicating a positive S-factor and stronger apparent strain stiffening in the intermediate strain range (Supplementary Fig. S1). The meat analogues products show less differences between the ${G}_{L}^{{\prime}}$, and ${G}_{M}^{{\prime}}$ upon deformation, exhibiting more mild apparent strain stiffening (Supplementary Fig. S2).

We conclude that meat products have a lower dissipation ratio and a higher S-factor than current meat analogues, especially after heating. It can be expected that the observed differences in elastic properties and stiffness will be lead to differences in sensory characteristics. For example, a previous study showed that the non-linear rheology of whey protein isolate/k-carrageenan gels correlated to sensory and oral processing. The ratio ${G}_{L}^{{\prime}}$/${G}_{M}^{{\prime}}$ positively and negatively correlated with oral processing data that including aspects after several chews as well as the first bite^25^. In other words, the non-linear rheological properties of meat and meat analogues can identify and quantify differences between meat and meat analogues.

Non-linear elasticity, and specifically strain-stiffening, is generic to filament/fibrous networks. The strain at which stiffening becomes significant depends strongly on the persistence length of the filament in a protein-based network. Stiffer filaments, like F-actin or collagen, stiffen at low strains whereas more flexible filaments stiffen only at larger strains^26^. The higher stiffening for meat products could be related to its unique hierarchical structure on small length scales. Future developments on meat analogues should therefore focus on routes how to create more stiffness and elasticity, for example through creating more physical and chemical interactions at mesoscopic length scale, thereby to some degree creating a hierarchical structure as well.

## Conclusion

We quantified the non-linear rheology resemblance of animal meat to their plant-based analogues by using a combination of texture maps and dissipation colour schemes. Meat analogues have similar stress and strain values at the end of the LVE regime and crossover point compared with heated meat, but their analogues are less elastic than heated (prepared) meat. In addition, the effect of heating on meat and meat analogues is different. Heating meat results in a tougher and more elastic material. For meat analogues there was only a small effect of a heat treatment observed, which is probably related to their processing history.

## Supplementary Information

Supplementary Information.
