# Supplementary material for: Non-linear rheology reveals the importance of elasticity in meat and meat analogues
Source: Sci Rep. 2022 Jan 25;12:1334. doi: 10.1038/s41598-021-04478-z (PMC8789867; doi:10.1038/s41598-021-04478-z)
Supplement: Supplementary file 1 — Supplementary Information. [file 41598_2021_4478_MOESM1_ESM.docx]

| Chicken | Beef | Pork |
| --- | --- | --- |
| At 30 °C | | |
|  |  | ●Gl’  ●Gm’ |
| Heated to 65°C and cooling to 30°C | | |
|  |  |  |
| Codfish | Salmon |  |
| At 30 °C | | |
|  |  |  |
| Heated to 65°C and cooling to 30°C | | |
|  |  |  |

Figure S1 The large strain modulus $G_{L}^{'}$ and zero strain modulus $G_{M}^{'}$ as function of strain for meat and fish at 30°C, and heated at 65°C and cooled to 30°C.

| Vegetarische basis wokstukjes | Kipstuckjes | Vivera plant stukjes als kip |
| --- | --- | --- |
| At 30 °C | | |
|  |  |  |
| Heated to 65°C and cooling to 30°C | | |
|  |  |  |
| Chick free pea based | Stukjes als van Kip |  |
| At 30 °C | | |
|  |  |  |
| Heated to 65°C and cooling to 30°C | | |
|  |  |  |

Figure S2 The large strain modulus $G_{L}^{'}$ and zero strain modulus $G_{M}^{'}$ as function of strain for meat analogues at 30°C, and heated at 65°C and cooled to 30°C.

Table S 1 Analysis of significances in differences of the stress and strain at the end of the LVE regime in Figure 2. Please note: Means with the same lower-case letter within a row (difference between temperature) were not significantly different (p<0.05). Means with the same capital letter within a column (difference between the products) were not significantly different (p<0.05).

|  | Comparing difference between products | | Comparing difference between temperature | | Comparing difference between products | | Comparing difference between temperature | |
| --- | --- | --- | --- | --- | --- | --- | --- | --- |
|  |  |  |  | |  |  |  | |
|  | Strain | Strain | Strain | Strain | Stress | Stress | Stress | Stress |
|  | 30°C | 65°C and cooled to 30°C | 30°C | 65°C and cooled to 30°C | 30°C | 65°C and cooled to 30°C | 30°C | 65°C and cooled to 30°C |
| Chicken | A | A | a | b | A | A | a | b |
| Beef | B | B | a | b | BC | A | a | b |
| Pork | C | C | a | b | BC | B | a | b |
| Codfish | D | C | a | b | C | C | a | b |
| Salmon | E | B | a | b | AB | BC | a | b |
| “Kipstuckjes” | F | D | a | b | D | AE | a | b |
| “Chick free pea based” | G | E | a | b | E | C | a | b |
| “Stukjes als van Kip” | D | F | a | b | F | D | a | b |
| “Vivera plant stukjes als kip” | B | G | a | a | G | E | a | a |
| “Vegetarische basis wokstukjes (AH)” | B | D | a | a | H | F | a | b |

Table S 2 Analysis of significances in differences of the stress and strain at the end of the LVE regime in Figure 3. Please note: Means with the same lower-case letter within a row (difference between temperature) were not significantly different (p<0.05). Means with the same capital letter within a column (difference between the products) were not significantly different (p<0.05).

|  | Comparing difference between products | | Comparing difference between temperature | | Comparing difference between products | | Comparing difference between temperature | |
| --- | --- | --- | --- | --- | --- | --- | --- | --- |
|  |  |  |  | |  |  |  | |
|  | Strain | Strain | Strain | Strain | Stress | Stress | Stress | Stress |
|  | 30°C | 65°C and cooled to 30°C | 30°C | 65°C and cooled to 30°C | 30°C | 65°C and cooled to 30°C | 30°C | 65°C and cooled to 30°C |
| Chicken | A | A | a | a | A | A | a | b |
| Beef | B | A | a | b | B | B | a | b |
| Pork | C | A | a | b | A | C | a | b |
| Codfish | D | A | a | b | BC | D | a | b |
| Salmon | D | A | a | b | C | D | a | b |
| “Kipstuckjes” | E | B | a | b | B | D | a | b |
| “Chick free pea based” | F | C | a | b | D | E | a | a |
| “Stukjes als van Kip” | F | C | a | b | D | E | a | a |
| “Vivera plant stukjes als kip” | A | D | a | b | E | EF | a | a |
| “Vegetarische basis wokstukjes (AH)” | D | E | a | b | F | F | a | a |
